# Homozygous R136S mutation in *PRNP* gene causes inherited early onset prion disease

**DOI:** 10.1186/s13195-021-00912-6

**Published:** 2021-10-18

**Authors:** Teresa Ximelis, Alba Marín-Moreno, Juan Carlos Espinosa, Hasier Eraña, Jorge M. Charco, Isabel Hernández, Carmen Riveira, Daniel Alcolea, Eva González-Roca, Iban Aldecoa, Laura Molina-Porcel, Piero Parchi, Marcello Rossi, Joaquín Castilla, Raquel Ruiz-García, Ellen Gelpi, Juan María Torres, Raquel Sánchez-Valle

**Affiliations:** 1 Neurological Tissue Bank of the Biobanc-Hospital Clinic-Institut d’Investigacions Biomediques August Pi i Sunyer (IDIBAPS), 08036 Barcelona, Spain; 2 Centro de Investigación en Sanidad Animal (CISA-INIA-CSIC), 28130 Valdeolmos, Madrid, Spain; 3 Center for Cooperative Research in Biosciences (CIC bioGUNE), Basque Research and Technology Alliance (BRTA), Bizkaia Technology Park, 48160 Derio, Spain; 4 Fundació ACE, Barcelona Alzheimer Treatment and Research Center, 08028 Barcelona, Spain; 5 Neurology Service, Hospital de León, 24071 León, Spain; 6 Memory Unit, Hospital de la Santa Creu i Sant Pau, 08041 Barcelona, Spain; 7 Immunology department, Biomedical Diagnostic Center, Hospital Clínic de Barcelona, 08036 Barcelona, Spain; 8 Pathology Department, Biomedical Diagnostic Center, Hospital Clínic de Barcelona, University of Barcelona, 08036 Barcelona, Spain; 9 Alzheimer’s Disease and Other Cognitive Disorders Unit, Neurology Service, Hospital Clínic de Barcelona, IDIBAPS, University of Barcelona, Villarroel, 170 08036 Barcelona, Spain; 10 Department of Experimental, Diagnostic and Specialty Medicine (DIMES), University of Bologna, 40138 Bologna, Italy; 11 IRCCS, Istituto delle Scienze Neurologiche di Bologna, 40139 Bologna, Italy; 12 IKERBasque Basque Foundation for Science, 48009 Bilbao, Spain; 13 Division of Neuropathology and Neurochemistry, Department of Neurology, Medical University of Vienna, 1090 Vienna, Austria

**Keywords:** Gene *PRNP*, GSS, Homozygous, Neuropathology, Prion

## Abstract

**Background:**

More than 40 pathogenic heterozygous *PRNP* mutations causing inherited prion diseases have been identified to date. Recessive inherited prion disease has not been described to date.

**Methods:**

We describe the clinical and neuropathological data of inherited early-onset prion disease caused by the rare *PRNP* homozygous mutation R136S. In vitro PrP^Sc^ propagation studies were performed using recombinant-adapted protein misfolding cyclic amplification technique. Brain material from two R136S homozygous patients was intracranially inoculated in TgMet129 and TgVal129 transgenic mice to assess the transmissibility of this rare inherited form of prion disease.

**Results:**

The index case presented symptoms of early-onset dementia beginning at the age of 49 and died at the age of 53. Neuropathological evaluation of the proband revealed abundant multicentric PrP plaques and Western blotting revealed a ~ 8 kDa protease-resistant, unglycosylated PrP^Sc^ fragment, consistent with a Gerstmann-Sträussler-Scheinker phenotype. Her youngest sibling suffered from progressive cognitive decline, motor impairment, and myoclonus with onset in her late 30s and died at the age of 48. Genetic analysis revealed the presence of the R136S mutation in homozygosis in the two affected subjects linked to homozygous methionine at codon 129. One sibling carrying the heterozygous R136S mutation, linked to homozygous methionine at codon 129, is still asymptomatic at the age of 74. The inoculation of human brain homogenates from our index case and an independent case from a Portuguese family with the same mutation in transgenic mice expressing human PrP and in vitro propagation of PrP^Sc^ studies failed to show disease transmissibility.

**Conclusion:**

In conclusion, biallelic R136S substitution is a rare variant that produces inherited early-onset human prion disease with a Gerstmann-Sträussler-Scheinker neuropathological and molecular signature. Even if the R136S variant is predicted to be “probably damaging”, heterozygous carriers are protected, at least from an early onset providing evidence for a potentially recessive pattern of inheritance in human prion diseases.

## Background

Genetic prion diseases represent ~ 15% of human prion diseases [1] and are caused by genetic variants in the *PRNP* gene, located on the chromosome 20. More than 40 rare variants have been described to cause genetic prion disease. Importantly, these mutations are linked to the polymorphism at codon 129, coding either for methionine or valine, which frequently determines the phenotypic presentation. Genetic prion diseases usually present family history of disease. However, in some genetic cases, the familial tree [2] could not identify other cases in the family or suggests incomplete penetrance, which has been attributed to underdiagnosis or low clinical expression during the lifespan [3, 4]. Mutations induce misfolding of the cellular prion protein (PrP^C^), and the accumulation of the disease associated isoform PrP^Sc^ [5, 6]. According to the clinic-neuropathological features, inherited prion diseases can be classified as genetic Creutzfeldt-Jakob disease, Gerstmann-Sträussler-Scheinker (GSS) syndrome, Fatal Familial Insomnia, and Prion diseases associated to octapeptide insertions although there are also some variants presenting an intermediate clinical phenotype or not fitting into these categories [7]. The clinical diagnosis of genetic prion disorders might be challenging, especially in patients with non-prototypical phenotypes. Analysis of the human *PRNP* gene is recommended whenever such as disease form is suspected [8, 9].

Up to date, all described pathogenic variants cause the disease in heterozygous carriers, being the disease inherited with an autosomal dominant pattern. Very few individuals have been reported to be homozygous for *PRNP* variants [10–13]. Most of them are homozygous carriers of well-known pathogenic mutations which cause also the disease in heterozygous carriers from areas of high prevalence of the mutation. In 2006, Pacheco et al. [14] reported at the Prion 2006 meeting a Portuguese patient carrying the homozygous R136S variant with family history of disease in two siblings, but not additional information is available in the literature.

In this report, we describe the clinical, neuropathological, and biochemical phenotype of genetic prion disease associated with a biallelic R136S substitution in the *PRNP* gene in a Spanish family. We also present the results of in vivo and in vitro transmissibility and propagation studies of this rare variant.

## Materials and methods

### Clinical data

The index case and two asymptomatic siblings were studied at the Alzheimer’s disease and other cognitive disorders unit at Hospital Clínic de Barcelona, Barcelona, Spain. Clinical information also included data from other centers specialized in cognitive disorders where the patient was evaluated and followed after diagnosis. The youngest sister and also an affected patient were examined at Hospital of Leon, Leon, Spain. The clinical information associated with other members of the family was obtained from medical records or was provided by the family members themselves.

### Genetic studies

*PRNP* exon 2 was amplified by PCR of genomic DNA extracted from whole peripheral blood as previously described [15] at Hospital Clínic de Barcelona. We used two pathogenic prediction softwares: SIFT [16] and Polymorphism Phenotyping v2 (Polyphen-2 (score 1.0)) [17] to predict the potential pathogenic effect of the observed variant. Copy number variation analysis was performed using Affymetrix SNP6.0 arrays.

### Neuropathology and immunoblotting

Neuropathologic examination was performed according to standardized protocols at Neurological Tissue Bank of Hospital Clínic-IDIBAPS in Barcelona, Spain. The right brain hemisphere was dissected in coronal sections and frozen while the left brain hemisphere was fixed by immersion in 4% formalin for 3 weeks and after fixation and cutting were treated with 98% formic acid for 1 h. Upon several water washes, brain material covering at least 30 different brain areas was postfixed in 10% formalin for at least 48 h and embedded in paraffin. For histologic evaluation, 5-μm-thick sections were stained with hematoxylin and eosin and periodic acid-Schiff (PAS). Immunohistochemistry (IHC) was performed using the Autostainer System method (Dako) with various antibodies including anti-PrP (12F10; Bertin-bioreagent, France), anti-βA4 (6F/3D, Dako, Glostrup, Denmark), anti-tau (AT8; Thermo Scientific, USA), anti-α-synuclein (5G4; Analytik Jena, Germany), anti-ubiquitin (P4D1; Cell signalling, USA), anti-α-internexin (2E3, Invitrogen, USA), and anti-TDP-43 (2E2-D3, Abnova, Taiwan). Disease evaluation was performed according to international consensus criteria [18].

Samples of frontal, temporal, parietal and occipital cortices, hypothalamus, thalamus, and cerebellum were processed for Western blotting. Human brain tissue was homogenized in 10% (w/v) in lysis buffer (100 mM NaCl, 10 mM EDTA, 0.5% Nonidet P-40, 0.5% sodium deoxycholate, 100 mM Tris) at pH 6.9. To detect brain PrP^res^ brain homogenate were subjected to digestion with 2 U/ml of proteinase K (PK) (Roche Diagnostics) at 37 °C for 1 h. After blocking PK activity with phenylmethylsulfonyl fluoride (PMSF, final concentration 3.6 mM), samples were boiled in sample buffer (final concentration: 3% SDS, 4% β-mercaptoethanol, 10% glycerol, 2 mM EDTA, 62.5 mM Tris) for 6 min at 100 °C. Digested samples were loaded into 12% Tris-Glycine gel (Criterion, Bio-Rad), after electrophoretic transference of proteins onto polyvinydene fluoride membranes (Bio-Rad) and after blocking the membrane 1 h at 37 °C. After electrophoretic transference of proteins onto polyvinylidene fluoride membranes (Bio-Rad) membranes were incubated with 3F4 (1:30.000; Millipore). 3F4 recognizes the 109-112 epitope of the human-PrP^C^ sequence. Immunocomplexes were detected by 1 h membrane incubation with horseradish peroxidase conjugated antimouse IgG (GE Healthcare Amersham Biosciences) and development with enhanced chemiluminescence in ECL Select (GE Healthcare Amersham Biosciences). For PrP^Sc^ deglycosylation, N-Linked glycans were removed by using a peptide-N-glycosidase (PNGaseF+) F kit (New England Biolabs) according to the manufacturer’s instructions.

### In vitro propagation of PrP by PMCA

In vitro PrP^Sc^ propagation studies were performed at the Prion Research Lab at CICbioGune, in Bizkaia, Spain. Two different types of experiments were performed using recombinant-adapted protein misfolding cyclic amplification (recPMCA) technique as previously described [19]. In the experiment focused on the in vitro propagation of human rec-PrP^Sc^ using R136 vs. S136 human rec-PrP (23-231) as substrates, samples were complemented with chicken brain homogenate and seeded with different dilutions of recPMCA-adapted CJD MM1 misfolded rec-PrP^Sc^ (seed obtained through serial passages in PMCA of an sCJD MM1 isolate on a substrate containing recombinant human M129 PrP), from 10^−1^ to 10^−8^ and subjected to a unique 24-h round of standard recPMCA. Specifically, the propagation capacity of this seed was evaluated in three different substrates human R136 rec-PrP, human S136 rec-PrP, and a mix (1:1) of both proteins, all of them coupled to M129 polymorphism. The second experiment, focused on the evaluation of the spontaneous propensity to misfold of the R136S variant, was performed twice using four replicates of four and compared with other human mutated rec-PrP (GSS-associated variants P105L and A117V) and wt rec-PrP. Samples were subjected to 15 serial rounds of recPMCA in the absence of seed and with dilution 1:10 in each round [20]. Amplified samples were digested with 50–80 μg/ml of PK and analyzed by Western blot using monoclonal antibody D18 (1:5000).

### Transmission studies in human PrP transgenic mice

Inoculation of transgenic mice was performed at Centro de Investigación en Sanidad Animal (CISA-INIA-CSIC), Madrid, Spain. Inocula were prepared from fresh frozen cerebellar tissue from the proband and from one independent case from the Portuguese family [14] carrying the homozygous R136S variant, as 10% (wt/vol) homogenates in 5% glucose distilled water. Two different mouse models for human-PrP were inoculated: HuPrP-Tg340-Met129 (TgMet129) mouse line expressing the Met129-PrP^C^ variant [21] and HuPrP-Tg362-Val129 mouse line expressing the Val129-PrP^C^ variant (TgVal129) [22]. These transgenic mouse lines express 4-fold and 8-fold the level of PrP^C^ expression in human brain respectively. Six to nine individually identified mice 6–10 weeks old were anesthetized and inoculated with 2 mg of 10% brain homogenate in the right parietal lobe by using a 25-gauge disposable hypodermic needle. After inoculation, mice were observed daily and their neurologic status was assessed weekly. At the established experimental endpoint (700 days postinoculation), animals were euthanized. After necropsy, part of the brain was fixed by using immersion in neutral-buffered 10% formalin (4% 2-formaldehyde) and used it for histopathology analysis while the rest of the tissue was frozen at − 20 °C and used for proteinase K–resistant PrP^Sc^ (PrP^res^) detection by WB. For each inoculation experiment, mean survival times with standard deviation was calculated as well as the attack rate defined as the proportion of mice that scored positive for PrP^res^ divided by the number of total inoculated mice.

### Western blotting in mouse bioassay

Western blotting analysis was done as previously described [23]. Briefly, 175 + 20 mg of frozen mouse brain tissue were homogenized in 5% glucose distilled water in grinding tubes (Bio-Rad) and adjusted to 10% (wt/vol) by using a TeSeE Precess 48TM homogenizer (Bio-Rad). To detect brain PrP^res^ 100 μL of 10% (wt/vol) brain homogenate were subjected to digestion with 40 μg/mL of PK in buffer 5% sarkosyl, 5% Triton X100, 1 M Urea, and 16 mM Tris–HCl (pH 9.6) at 60 °C for 15 min. Digested samples were loaded samples into 12% Bis-Tris Gel (Criterion XT; Bio-Rad). After electrophoretic transference of proteins onto polyvinylidene fluoride membranes (Millipore) and blocking overnight with 2% bovine serum albumin blocking buffer, membranes were incubated with 12B2 [24] and Sha31 [25] mAb at a concentration of 1 μg/mL. 12B2 recognizes the 89-WGQGG-93 epitope of the human-PrP^C^ sequence while Sha31 recognizes the 145-WEDRYYRE-152 epitope of the human-PrP^C^ sequence. Immunocomplexes were detected by 1 h membrane incubation with horseradish peroxidase conjugated antimouse IgG (GE Healthcare Amersham Biosciences) and development with enhanced chemiluminescence in ECL Select (GE Healthcare Amersham Biosciences). Images were captured using the ChemiDoc WRS+ System and processed them by using Image Lab 5.2.1 software (both Bio-Rad).

### Histological analysis in mouse bioassay

Mouse brain samples were immediately fixed in neutral-buffered 10% formalin (4% 2-formaldehyde) during necropsy, coronally trimmed, and processed routinely. After embedded in paraffin-wax, tissues were cut to 4-μm thickness, de-waxed, and rehydrated by standard procedures. Tissue slides were subjected to two different histological methods: hematoxylin and eosin staining for lesion profile evaluation by standard methods [26] and IHC. Lesion profile evaluation was done by semi-quantitative assessment of spongiform degeneration scoring vacuolization in the following brain areas: cerebral cortex, striatum, hippocampus, thalamus, hypothalamus, midbrain, cerebellar cortex, and pons/medulla oblongata. For the IHC analysis, tissue slides were subjected to antigen retrieval and quenching of hydrogen peroxide, latter incubated with the Sha31 mAb [25] and subsequent steps of the IHC procedure were performed by a commercial immunoperoxidase technique (Vector-Elite ABC kit, Vector Laboratories) as per manufacturer’s instructions finally counterstaining the sections with Harri’s hematoxylin.

## Results

### Clinical characterization and family history

**Fig. 1:**
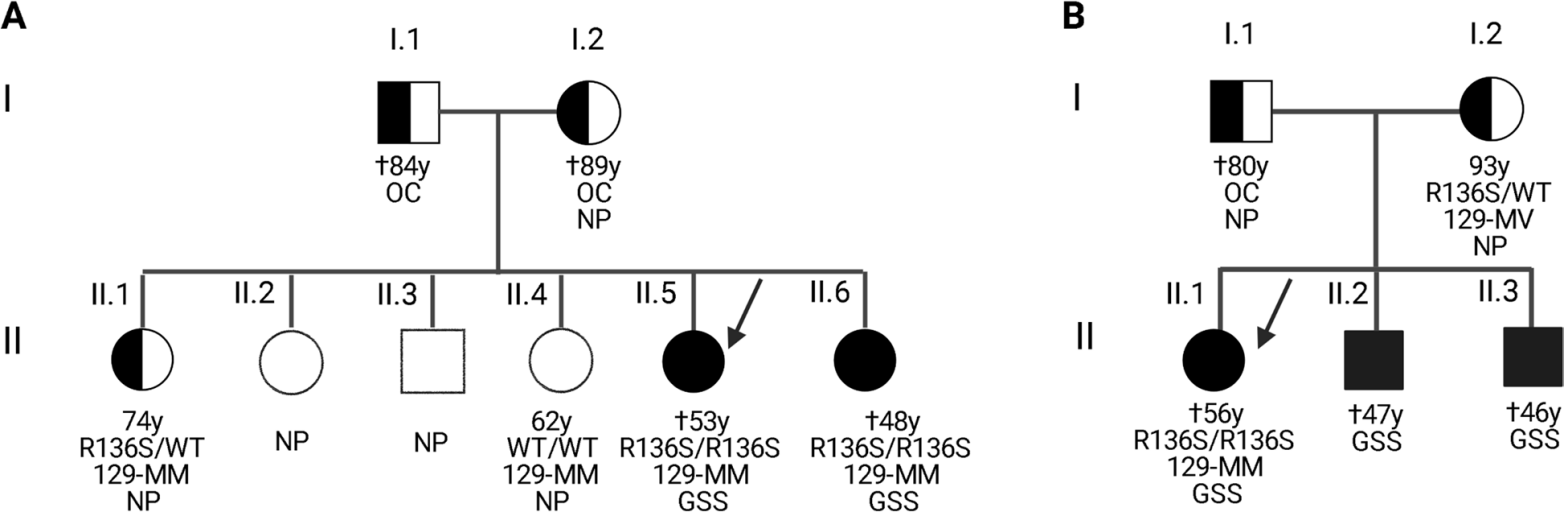
Genetic pedigrees. **A** Visual representation of the R136S Spanish family history. **B** Visual representation of the homozygous R136S Portuguese family (inferred from Pacheco et al. [14]). Created with BioRender.com

**Fig. 2:**
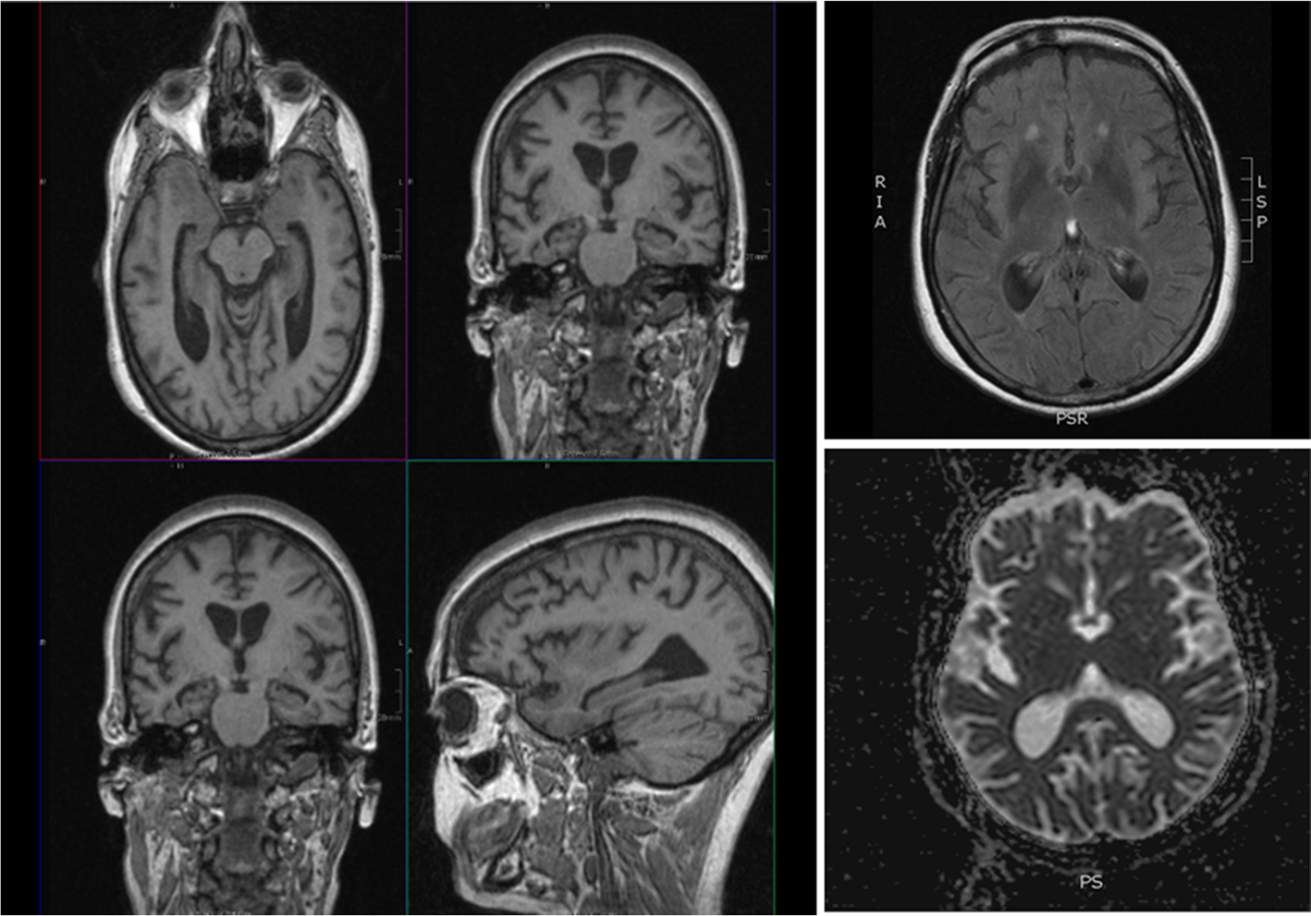
Magnetic resonance imaging scan of the index patient

The index patient was a 51-year-old female who was referred to the Neurology department due to progressive memory decline, language problems, and subtle changes in behavior (apathy and childish behavior) beginning at the age of 49 years (Table 1 and Fig. 1A, subject II.5). At first examination, her Minimental State Examination Score was 28/30. The cognitive evaluation revealed moderate generalized cognitive impairment including mild verbal memory impairment and visuospatial, language, and executive disturbances. The neurological examination was normal except for mild dysarthria. Nerve conduction studies were not performed. MRI examination was unremarkable (Fig. 2). Cerebrospinal fluid showed elevated total tau levels (1262 pg/ml), Abeta42 (701 pg/ml) within the normal range, slightly elevated phospho-tau (68.5 pg/ml), and reduced p-tau/t-tau ratio (0.054). The 14-3-3 Western blot test was negative. The patient deteriorated progressively over the next years with worsening of both cognitive and motor functions developing severe ataxia. At the last neurological examination, the patient presented severe inattention, only partial response to verbal stimuli, mutism, and generalized hypertonia and hyperreflexia and clonus in lower extremities. She died at the age of 53 after disease duration of 4 years.

**Table 1:** Demographic and clinical features of homozygous PRNP mutations in the literature

	Present study					
	Spanish family	Portuguese family [14]	Beck et al [11]	Komatsu et al [12]	Simon et al [10]	Hassan et al [27]
**Gender**	2 female	2 male/1 female	Female	Female	3 male/2 female	Male
**Country**	Spain	Portugal	Ireland	Japan	Libya	China
**Family history**	Yes	Yes (siblings)	No (grandmother with dementia in later life)	No	Yes	Unknown
**PRNP mutation**	R136S	R136S	Q212P	V203I	E200K	E200D
**Polymorhism codon 129**	MM	MM	MM	MM	Unknown	MM
**Phenotype**	Gerstmann-Sträussler-Scheinker	Gerstmann-Sträussler-Scheinker	Unknown	genetic Creutzfeldt Jakob’s disease	genetic Creutzfeldt Jakob’s disease	Unknown
**Cosanguinity**	Yes	Unknown	Probable	Yes	Unknown	Probable
**Age at onset (years)**	43.5 ± 5.5	52 (female)	36	73	50.4 ± 6.2	61
**Age of death (years)**	50.5 ± 2.5	49.6 ± 6.4	Unknown	75	50.8 ± 8.2	61
**Disease duration**	7.25 ± 2.75 years	4 years	4 years	2 years	15.8 ± 15.9 years	10 weeks
**Clinical symptoms**	Progressive memory decline (2/2), language (1/2), executive disturbances (2/2)	Psychiatric symptoms, myoclonous, Parkinsonian symptoms, and progressive dementia	Dysarthria, ataxia, nystagmus, and executive dysfunction	Gait disturbance, cognitive dysfunction	Dementia (5/5), personality change (2/5), parkinsonism (4/5); myoclonus (2/5), gait ataxia (2/5), and dysarthria (3/5)	Confusion, dysarthria, ataxia, myoclonus, and hallucinations
**14-3-3 protein**	Negative	Negative	Unknown	Positive	Unknown	Negative
**MRI brain**	Normal	Normal	Moderate cerebral atrophy	Hyperintensity in basal ganglia and right frontal, parietal and occipital lobes	Unknown	Abnormal symmetric restricted bilateral diffusion in the striatum and cortex
**EEG**	Unknown	Lentified	Mildly abnormal	Diffuse slowing of waves	3/5 abnormal	Mild slowing only, with no periodic sharp ware complexes
**Neuropathology**	Multicentric amyloid PrP plaques, prominent neuronal loss, astrogliosis, and microglial proliferation	Multicentric amyloid PrP plaques, mild neuritic component, and microglial activation	N/A	N/A	1/2 did not display PrP^res^	N/A
**PrP Western blotting**	~ 8 kDa band	8 kDa and 5 kDa band	N/A	N/A	N/A	N/A
**In vivo transmissibility studies**	Negative	N/A	N/A	N/A	N/A	N/A
**In vitro propagation studies**	Negative	N/A	N/A	N/A	N/A	N/A

The youngest sibling of the index patient (Table 1 and Fig. 1A subject II-6) also suffered from cognitive decline, motor impairment, and myoclonus with onset in her late 30s (38 years). Neurological examination, MRI scan, and CSF 14-3-3 were normal at first examination. At the age of 45, the neurological examination disclosed a MMSE score of 24/30, dysarthria, apraxia, gait disorder with short steps, generalized bradykinesia, hypertonia, and brisk reflexes. No sensory problems were observed. At the age of 46, she presented akinetic mutism, diffuse myoclonus, and severe generalized hypertonia. The patient died at the age of 48, but the postmortem neuropathological examination was not performed. The other four siblings were neurologically preserved (present ages from 63 to 74 years) (Fig. 1A, II.1–II.4).

The proband’s parents were born in a small village in the North-west of Spain and were probably consanguineous but the degree of kinship is unknown. The mother (Fig. 1A, subject I.2) died at the age of 89 years and was neurologically healthy according to their family members. The father (Fig. 1A, subject I.1) died at the age of 84 with dementia with a clinical onset at the age of 75 that had been classified as vascular cognitive impairment by the treating physician.

### Genetic studies

The genetic study of the Spanish patient and her youngest sister showed the presence of a substitution of guanine for thymine (AGG to AGT) at codon 136 of the *PRNP* in the two alleles, causing the missense mutation R136S in homozygosis. This was linked to methionine homozygosis at codon 129. No deletions or duplications were detected in the 100-kb region which includes the *PRNP* gene.

Of the four asymptomatic siblings, two underwent genetic testing. One asymptomatic 74-year-old sibling (Fig. 1A, subject II.1) carried the R136S mutation in heterozygosis, which was also linked to homozygous methionine at codon 129. Another sibling showed a normal *PRNP* sequence and was also methionine homozygous at codon 129.

According to pathogenic prediction software, the R136S variant is considered to be damaging (SIFT) [16] or probably damaging (Polymorphism Phenotyping v2 (Polyphen-2 (score 1.0)) [17].

### Neuropathological findings and immunoblotting

**Fig. 3:**
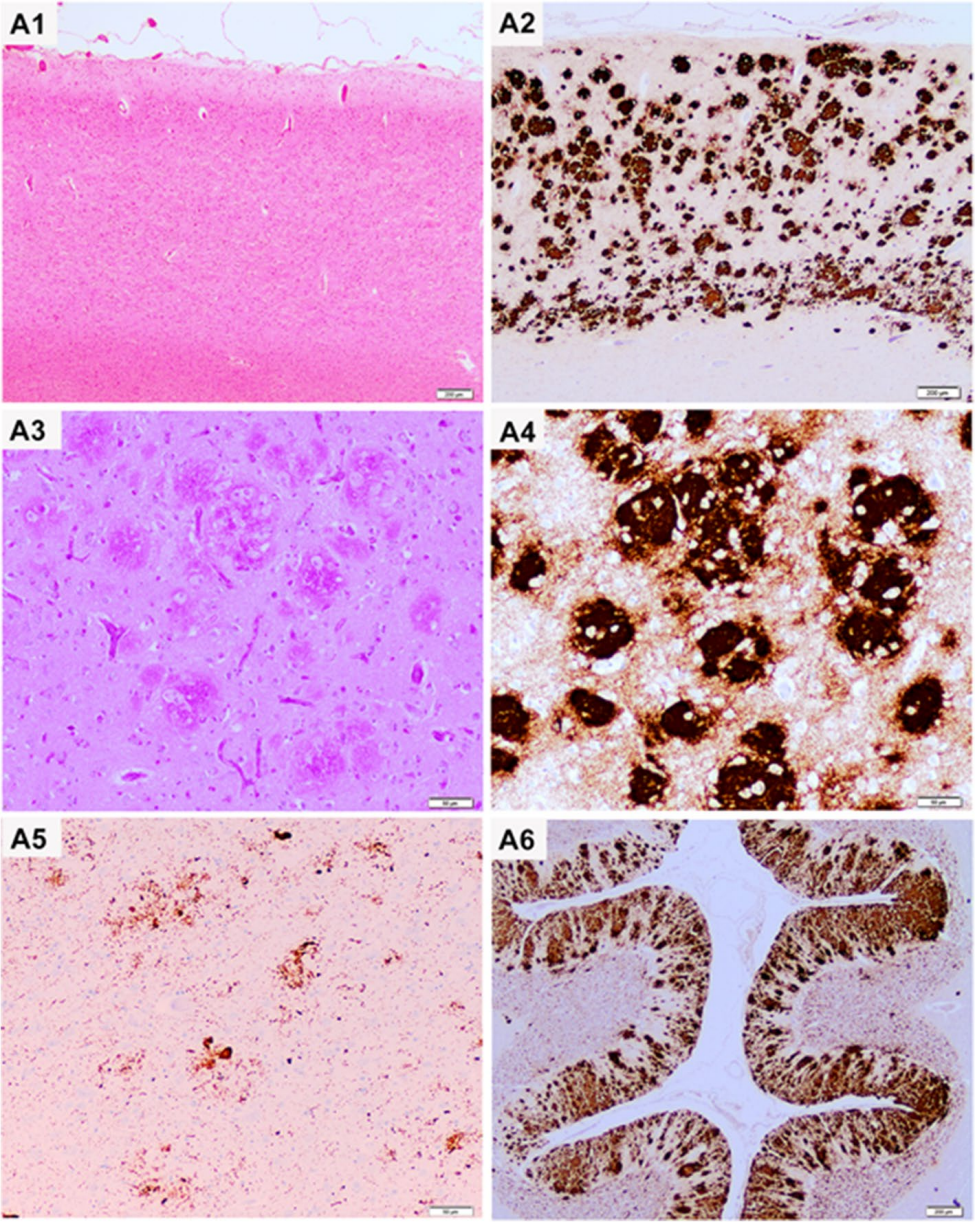
Neuropathological characterization. **A1** Frontal cortex shows prominent neuronal loss and gliosis with loss of cortical structure and mild superficial spongiosis (H&E stain). **A2** Anti-PrP immunohistochemistry (12F10 antibody) shows a very high density of multicentric PrP plaques covering the whole cortical thickness. **A3** These large multicentric plaques are well identified in the PAS stain and the clusters of large amyloid plaques are observed by immunohistochemistry with the 12F10 anti-PrP antibody (**A4**). **A5** Immunohistochemistry for hyperphosphorylated tau shows marked dystrophic neurites surrounding the plaques in the hippocampus (AT8 immunohistochemistry). **A6** Abundant multicentric plaques are detected in the molecular layer of the cerebellum (anti-PrP immunostaining, 12F10 antibody)

The brain of the index patient was removed after death at the Neurological Tissue Bank of the IDIBAPS Biobank in Barcelona. Unfixed brain weight was 1030 g. Gross examination showed a moderate diffuse brain atrophy accentuated in the frontal lobes and mild nigral pallor. Histology revealed a severe involvement of grey matter that showed prominent neuronal loss, astrogliosis, and microglial proliferation, especially in frontal cortex (Fig. 3A). Despite observing a superficial laminar spongiosis, only mild bona fide spongiform change was detected in grey matter which was characterized by a few small-sized vacuoles in cortical regions and basal ganglia. The most striking feature was the presence of abundant amyloid plaques consisting of multiple densely packed cores (multicentric plaques) that were variably PAS positive and strongly immunoreactive for PrP^Sc^ (12F10 antibody) after pretreating the tissue sections for removal of physiological PrP^C^. These multicentric plaques were detected in all cortical areas — with less involvement of primary visual cortex, in basal ganglia, thalamic nuclei, and brainstem and in the molecular layer of cerebellar cortex. Moreover, multicentric PrP amyloid plaques induced a moderate neuritic component that was immunoreactive for phosphorylated tau (AT8).

**Fig. 4:**
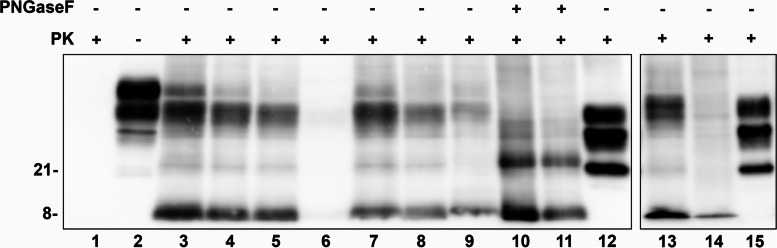
Western blot PrP^resR136S^ profile in fresh-frozen tissue of the index patient. In contrast to typical sCJDMM1 (lanes 12 and 15), PK-treated tissue homogenates from different brain regions (lanes 3–9: frontal cortex (3), temporal cortex (4), parietal cortex (5), occipital cortex (6), hippocampus (7), thalamus (8), cerebellum (9)) of the index patient show the association of a low molecular weight fragment of ~ 8 kDa with less defined upper bands in the ~ 21–30 kDa range. After de-glycosylation with PNGase F (lanes 10 and 11), the ~ 8 kDa is unchanged. In contrast, the upper bands merge in a single band of ~ 21 kDa migrating slightly above the unglycosylated fragment of PrPSc type 1 detected in sCJDMM1. Lanes 1 and 2 show a brain homogenate from a control with (1) or without (2, PrP^C^ profile) PK digestion. The comparison between the PrP^Sc^ profiles of the index patient and a GSS patient carrying the P102L mutation, in which only the hallmark ~ 8 kDa peptide is seen, is shown in lanes 13 and 14

PrP^Sc^ typing by Western blotting displayed the association of a low molecular weight (mw) fragment of ~ 8 kDa with less defined upper bands in the ~ 21–30 kDa range (Fig. 4A, B). After de-glycosylation with PNGase F, the ~ 8 kDa remained unchanged whereas the upper bands merged in a single band of ~ 21 kDa, migrating slightly above the unglycosylated fragment of PrP^Sc^ type 1 detected in CJD (Fig. 4A). The relative intensity of the upper and lower fragments varied between different brain regions. At the visual inspection, the frontal cortex contained larger amounts of PrP^Sc^.

### In vitro propagation of PrP^Sc^

**Fig. 5:**
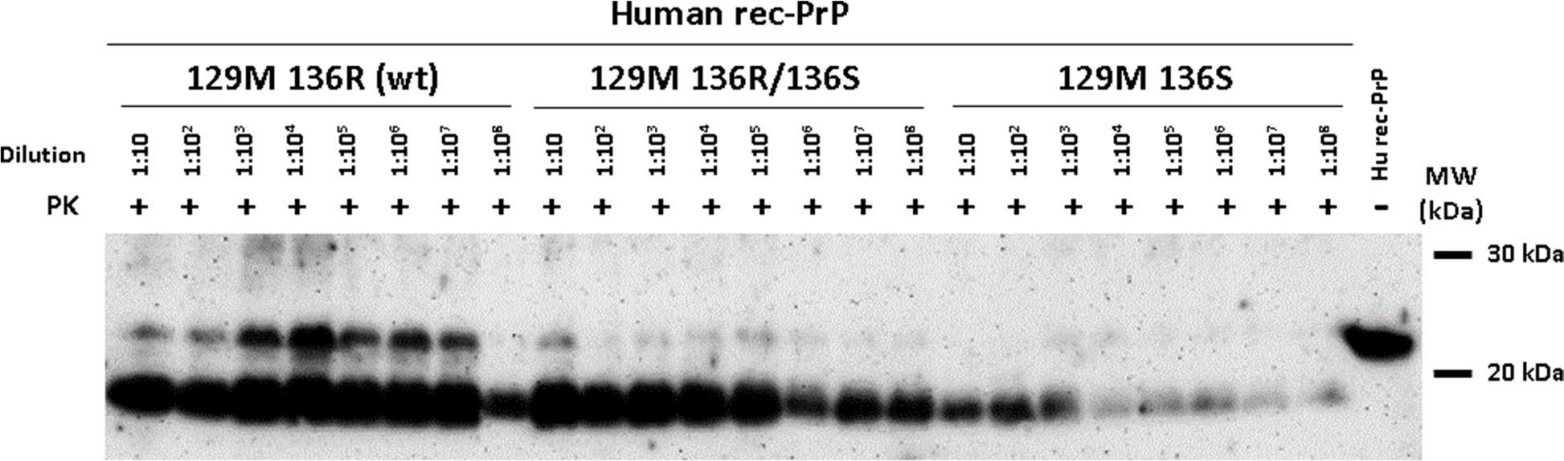
In vitro human misfolded PrP propagation by recPMCA using R136 vs. S136 human as substrates. Human 129M 136R (wt) rec-PrP, human 129M 136S rec-PrP, and a mix of both rec-PrPs in ratio 1:1 were complemented with chicken brain homogenate, seeded with different dilutions of recPMCA-adapted CJD MM1 misfolded rec-PrP (10-1–10-8) and subjected to a unique 24 h round of standard recPMCA. Amplified samples were digested with 50 μg/ml of Proteinase-K and analyzed by Western blot using monoclonal antibody D18 (1:5000). No significant differences were found in the propagation ability of the R136 (wt) vs. the mutant R136S rec-PrP or the mix 1:1 of both proteins

In order to evaluate the effect of the R136S mutation in the in vitro propagation of human rec-PrP^Sc^, human 129M 136R (wt) rec-PrP, human 129M 136S rec-PrP, and a mix of both rec-PrPs in ratio 1:1 were used as substrates for recPMCA and seeded with different dilutions of recPMCA-adapted CJD MM1 misfolded rec-PrP (10-1–10-8), and subjected to a unique 24-h round of standard recPMCA. No significant differences were found in the propagation ability of the R136 (wt) vs. the mutant R136S rec-PrP or the mix 1:1 of both proteins (Fig. 5).

**Fig. 6:**
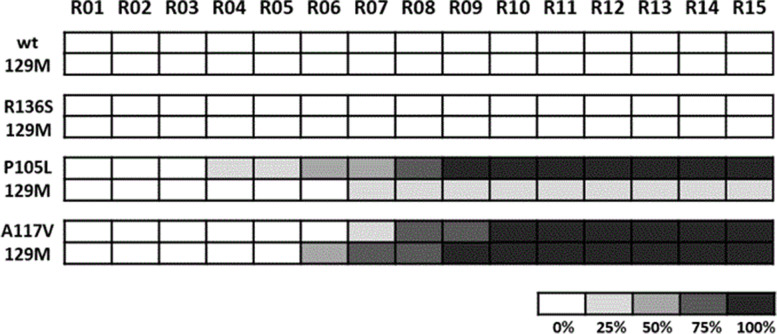
Spontaneous generation of human misfolded recombinant proteins. Graphical representation of the emergence of spontaneous protein misfolding evaluated through Western blot analysis in SDS-PAGE for each round of recPMCA (outlined as R01–R15). Different 129M human recombinant proteins were grouped according to the substitution. Every experiment contained four tubes (intra-experimental duplicates) and was performed in duplicate as shown. The percentage of positive tubes (tubes showing a protease resistant signal after digestion with 80 mg/ml of PK) after each round of recPMCA was noted with different intensities of grey, as shown in the legend below the figure. Neither wild-type (wt) 129M not R136S 129M were able to misfold spontaneously. WT, wild type human rec-PrP^Sc^

To evaluate the spontaneous propensity of the R136S recPrP to misfold compared to the wt recPrP and other GSS-related mutated recPrP (P105L and A117V), a serial rec-PMCA (15 rounds) was performed in the absence of PrP^Sc^ seed and performing 1:10 dilutions of the previous passage after each round (Fig. 6). All cases were associated with methionine at codon 129. The wt and the R136S PrP linked to the methionine polymorphic variant at codon 129 did not show spontaneous misfolding, in contrast to the other GSS-related recPrP.

### Bioassay in human-PrP transgenic mice

**Fig. 7:**
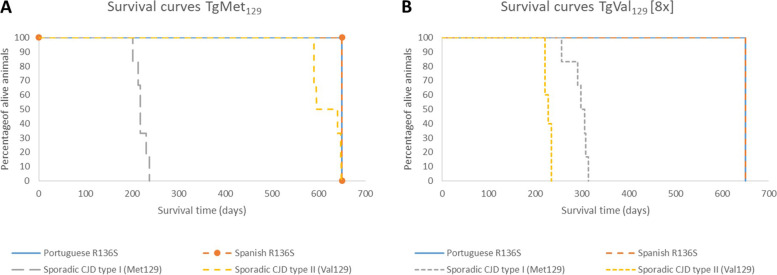
Visual representation of the survival curves in the transgenic mouse lines **A** TgMet_129_ and **B** TgVal_129_

Brain material from the two patients (the Spanish index case and the Portuguese index case) were intracranially inoculated in TgMet129 and TgVal129 transgenic mice (26) to assess the transmissibility of this rare inherited form of prion disease (Table 2). These mouse lines expressed in homozygosis the Met129-PrP^C^ variant or the Val129-PrP^C^ variant respectively. All mice were sacrificed at the end of their lifespan without showing any clinical sign suspicious of prion disease and remaining uninfected (Fig. 7). No PrP^res^ was found in mice brains either by WB or IHC (data not shown). Vacuolization was no different from the one associated with the natural aging process of non-inoculated control mice of the same transgenic lines by histology (data not shown).

**Table 2:** Transmission of Spanish and Portuguese R136S cases in TgMet129 and TgVal129 [8x] mice

Inocula	Mice survival (attack rate) ST^**a**^ (n/n0)^**b**^	
	TgMet129	TgVal129 [8x]
**Portuguese R136S case**	> 650 days (0/5)	> 650 days (0/5)
**Spanish R136S case**	> 650 days (0/5)	> 650 days (0/5)
**Sporadic CJD type I (Met**^**129**^**)**	219 ± 17 (6/6)^c^	295 ± 21 (6/6)^d^
**Sporadic CJD type II (Val**_**129**_**)**	618 ± 81 (6/6)^c^	228 ± 7 (5/5)^d^

^a^Mean survival time

^b^Attack rate was calculated as the relation between brain PrP^res^ positive animals (= *n*) and total number of inoculated animals (= *n*0)

^c^Previously published in Cassard et al. [28], Fernández-Borges et al. [29], and Marín-Moreno et al. [30]

^d^Previously published in Fernández-Borges et al. [29]

## Discussion

We report here that the biallelic R136S *PRNP* mutation causes inherited human prion disease with early onset of symptoms (30s–40s). The same mutation presented in heterozygous state in other family members did not induce the same phenotype. Clinically, the patients presented with early-onset dementia developing motor disturbances at follow-up. Neuropathological examination revealed the presence of abundant multicentric PrP amyloid plaques with mild neuritic component, along with severe neuronal loss, gliosis, and microglial proliferation with only mild spongiform change, which is consistent with a GSS neuropathological phenotype. In contrast to previous description of homozygous mutations in *PRNP* [10–13, 27, 31] (Table 1), the disease in R136S families presented with an autosomal recessive pattern of inheritance, keeping the proven or obligated heterozygous family members protected from developing the disease, at least, at early ages. In vitro propagation and in vivo transmissibility studies suggest that the PrP^Sc^ variant resulting from the R136S mutation failed to propagate both in vivo and in vitro in the conditions analyzed, suggesting that this variant is biologically less active than other mutations causing genetic prion diseases.

Previous described pathogenic mutations cause human prion disease in the heterozygous state and show an autosomal dominant inheritance pattern. There are scarce examples in the literature of patients affected by prion diseases carrying homozygous genetic variants. Homozygous E200K carriers are associated with consanguinity in areas of high prevalence of this variant. Homozygous carriers of the E200K mutation present an earlier age of onset than heterozygous carriers but no other relevant differences in clinical features compared with heterozygous carriers [10]. One case of prion disease linked to homozygous Q212P has been described, but also another case has been reported in a heterozygous carrier [11]. Komatsu et al. [12] reported the case of a homozygous V203I patient, in addition to three previously reported heterozygous patients for this variant. However, the lack of family history of disease in all the V203I and the presence of this variant in normal controls causes that its pathogenicity is still under discussion [3, 11, 31]. Recently, Hassan et al. [27] also reported a case with CJD and a homozygous E200D mutation, similar to Q212P [11].

Minikel et al. [3] reported in 2016 the presence of the R136S variant in heterozygosis in two alleles from 60.706 population control exomes but not in patients, suggesting that this represents a very rare variant in the normal population even if the R136S variant is predicted to be “probably damaging” in several pathogenicity prediction software. In our center, we had not identified this genetic variant in more than 300 Spanish subjects (including both patients and controls) analyzed so far. In 2006, Pacheco et al. [14] described one Portuguese patient (Fig. 1B, subject II.1) with similar clinical and neuropathological features of those of our patients (early-onset dementia, motor symptoms in her early 50s and a GSS neuropathological phenotype) linked to the presence of the homozygous R136S variant. Two of her siblings presented history of similar disease in their early 50s, but not the parents who should be obligated carriers.

In both families (the present family and the Portuguese family) described heterozygous carriers were apparently protected from disease. As the genetic study in the Portuguese index case’s mother (Fig. 1B, subject I.2) and another family member neurologically preserved in their 80s and 90s revealed the presence of heterozygous R136S linked to 129MV, the authors suggested that the presence of valine in the trans-allele could prevent the expression of the disease. In contrast, in the Spanish family, one heterozygous carrier, homozygous for methionine at codon 129 is neurologically preserved in their 70s, suggesting that the expression of the disease does not depend on the codon 129 of the trans-allele but in the homozygous state or “dosage” of the mutant allele.

The expression of the disease only in homozygous subjects could be explained by a dose effect as it has been suggested for an earlier age of onset in homozygous E200K carriers [10]. A larger number of R136S PrP molecules (or the absence of wild-type protein) in the homozygous patient’s brain may increase the chance of spontaneous conversion. Even if homozygous patients are at higher risk for the spontaneous conversion to PrP^Sc^ than heterozygous patients, on the other hand, the subsequent infective process based on PrP^Sc^-PrP^C^ interaction might be less effective as there is only mutant PrP^Sc^ leading to longer disease duration.

Even if we cannot rule-out a late onset of the disease, the presence of known or obligated heterozygous carriers in neurological preserved family members that were already in their 70s, 80s, or 90s make this possibility much less probable during the usual lifespan. In this sense, we believe that both family trees (Fig. 1) provide evidence for a recessive pattern of inheritance in human prion diseases.

The clinical phenotype in the patients resembled that of insertion mutations (OPRI), which are characterized by early-onset progressive dementia with late motor dysfunction [6], whereas the neuropathological features were typical of GSS. The observed clinical phenotype is atypical for GSS, which more commonly present with motor signs followed by dementia, although it has also been linked to other point mutations (Y218N) [6] and OPRI [31, 32]. Irrespectively of the clinical syndrome and the relative regional distribution of lesions, GSS is defined by the presence of multicentric PrP amyloid plaques in the cerebral and cerebellar cortices [7, 18]. In contrast to other human prion diseases, the presence of spongiform change can be variable, from absent to severe, which could explain the lack of signal intensity changes in diffusion-weighted imaging in MRI scans. In addition, Western blot studies typically show, as in the present case, PK-resistant non-glycosylated PrP^Sc^ fragments with a low molecular weight that varies between 6 and 8 kDa depending on the PRNP pathogenic variant. Occasionally, however, the immunoblot profile also shows larger truncated PrP^res^ fragments with a molecular weight of 19–21 kDa, as observed in other sporadic or genetic prion diseases [33–36].

Animal modeling of human inherited prion diseases is not easy. Practically all attempts to generate transgenic mouse models for human inherited prion diseases using the human-PrP^C^ sequence had been unsuccessful apart from the recent reported exception of an A117V GSS model [37]. The rest of successful transgenic mouse models that develop spontaneously neurologic disorders when harboring mutations that have been reported in human inherited prion diseases have been made in mouse or bank vole PrP sequences or in chimeric mouse/human PrP proteins [38]. It seems that mouse and bank vole PrP proteins are more prone to spontaneous misfolding while human PrP is apparently more resistant. Alternatively, interactions between mouse PrP and other than PrP mouse factors may be important for the spontaneous generation of prions. In general terms for GSS modelling, models overexpressing mouse, mouse/human chimeric, and cow PrP harboring the respective equivalents to human P102L mutation spontaneously developed a prion disease along with neuropathological changes [38]. However, brain PrP^res^ detection and transmissibility to wild-type mice was only achieved in the particular case of equivalent P113L mutation in cow-PrP^C^ sequence producing a prion agent with features resembling those of classical bovine spongiform encephalopathy [39]. When the A117V mutation was overexpressed in the mouse sequence (A116V) at 4–6x levels, transgenic mice showed signs of prion disease including neuropathology markers but no PrP^res^ were detected [40]. However, A117V GSS cases were successfully transmitted into A117V human PrP transgenic mice Tg30 and Tg31 (2x and 3x overexpression respectively) [37, 41] from which Tg30 developed a spontaneous disease that was later transmissible to both Tg30 and Tg31 mice as well as to wild-type V129 human PrP transgenic mice [37]. It would be of interest to further investigate the effects of this homozygous mutation in an animal model, as well as the production of heterozygous animals to further investigate to which extent the presence of the wild-type allele dumpers the spontaneous misfolding of the mutated one. The recessive inheritance pattern displayed by this disease in humans points to a protective effect exerted by the wild-type variant and the in vitro studies performed in this work also supports this hypothesis. However, a slower misfolding rate of the wild-type allele that may finally contribute to disease development at elder ages cannot be ruled out.

The in vitro propagation and spontaneous misfolding proneness studies, performed by recPMCA, indicate that the R136S mutation does not confer an enhanced misfolding propensity to the human recombinant PrP. No differences were detected on misfolding proneness of this protein compared to the wild type, neither induced or spontaneously. In fact, prion ability to cause direct cellular damage (toxicity) has often been dissociated from the capacity of successfully transmit from an infected host to an uninfected recipient (transmissibility) [42]. This, added to the difficulty of modelling inherited prion disease that is further discussed below, may explain the lack of in vivo and in vitro propagation of the prion disease associated to R136S mutation. The synthesis of peptides containing the mutation and testing their effect on cultured cells, apart from allowing determining differences in the misfolding proneness of mutated proteins as the PMCA assays carried out here, could allow evaluating potential changes in their neurotoxic properties [43].

### Limitations

This report has some limitations. First, the diagnosis of genetic prion disease is a hurdle task and some members of both extended families might have been underdiagnosed, lowering the possibilities of describing this mutation in other patients. Second, due to the lack of neuropathological evaluation in any of the asymptomatic heterozygous carriers, we could not rule-out subclinical prion disease at late ages, although this possibility seems less probable.

## Conclusions

The biallelic R136S substitution is a rare *PRNP* mutation that induces inherited human prion disease presenting with early-onset dementia and motor impairment and neuropathological and molecular features consistent with GSS. Even if the R136S variant is predicted to be “probably damaging”, heterozygous carriers are protected, at least, from an early-onset, providing evidence for a potentially recessive pattern of inheritance in human prion diseases.

## Data Availability

The datasets used and/or analyzed during the current study are available from the corresponding author on reasonable request.

## Abbreviations

PrP^C^: Cellular prion protein
GSS: Gerstmann-Sträussler-Scheinker
TgMet129: HuPrP-Tg340-Met129
IHC: Immunohistochemistry
NP: Neurologically preserved
OP: Obligated carrier
PAS: Periodic acid-Schiff
PNGaseF+: Peptide-N-glycosidase
PMSF: Phenylmethylsulfonyl fluoride
recPMCA: Protein misfolding cyclic amplification
PK: Proteinase K
PrP^res^: Proteinase K–resistant PrP^Sc^
TgVal129: Val129-PrP^C^ variant
